# Prostate apoptosis response-4 is involved in the apoptosis response to docetaxel in MCF-7 breast cancer cells

**DOI:** 10.3892/ijo.2013.1983

**Published:** 2013-06-12

**Authors:** MICHELLY C. PEREIRA, SIMONE A. DE BESSA-GARCIA, RAVSHAN BURIKHANOV, ANA CAROLINA PAVANELLI, LOURIVAL ANTUNES, VIVEK M. RANGNEKAR, MARIA A. NAGAI

**Affiliations:** 1 Discipline of Oncology, Department of Radiology and Oncology, Faculty of Medicine, University of São Paulo, CEP 01246-903, São Paulo;; 2 Laboratory of Molecular Genetics, Center for Translational Research in Oncology, CEP 01246-000, São Paulo, Brazil;; 3 Markey Cancer Center, University of Kentucky, Lexington, KY, USA

**Keywords:** Par-4, breast cancer, proliferation, survival, apoptosis, docetaxel

## Abstract

Experimental evidence indicates that prostate apoptosis response-4 (Par-4, also known as PAWR) is a key regulator of cancer cell survival and may be a target for cancer-selective targeted therapeutics. Par-4 was first identified in prostate cancer cells undergoing apoptosis. Both intracellular and extracellular Par-4 have been implicated in apoptosis. Relatively little is known about the role of Par-4 in breast cancer cell apoptosis. In this study, we sought to investigate the effects of Par-4 expression on cell proliferation, apoptosis and drug sensitivity in breast cancer cells. MCF-7 cells were stably transfected with expression vectors for Par-4, or transiently transfected with siRNA for Par-4 knockdown. Cell proliferation assays were performed using MTT and apoptosis was evaluated using acridine orange staining, fluorescence microscopy and flow cytometry. Par-4 overexpression reduced MCF-7 proliferation rates. Conversely, Par-4 knockdown led to increased MCF-7 proliferation. Par-4 downregulation also led to increased BCL-2 and reduced BID expression. Par-4 overexpression did not affect the cell cycle profile. However, MCF-7 cells with increased Par-4 expression showed reduced ERK phosphorylation, suggesting that the inhibition of cell proliferation promoted by Par-4 may be mediated by the MAPK/ERK1/2 pathway. MCF-7 cells with increased Par-4 expression showed a marginal increase in early apoptotic cells. Importantly, we found that Par-4 expression modulates apoptosis in response to docetaxel in MCF7 breast cancer cells. Par-4 exerts growth inhibitory effects on breast cancer cells and chemosensitizes them to docetaxel.

## Introduction

Breast cancer is the most frequently diagnosed cancer in the industrialized world (1). Like other tumors, breast tumorigenesis involves the accumulation of multiple genetic and epigenetic changes affecting mechanisms that control cell proliferation, survival and apoptosis. The balance between proliferation, differentiation and cell death is critical for mammary gland development and maintenance (2). The ability of cancer cells to evade apoptosis is one of the essential alterations in cell physiology that dictate tumor growth and impact chemo- and radio-resistance (3,4).

Prostate apoptosis response-4 (Par-4), also known as PAWR, was first identified as upregulated in rat prostate cells undergoing apoptosis (5). Par-4 encodes a ubiquitously expressed pro-apoptotic protein that is localized to the cytoplasm of diverse normal tissues and cell lines; it is present in both the cytoplasm and the nucleus of many tumors and cancer cells (6–8). Endogenous Par-4 itself does not cause cell death, although it is essential for apoptosis induced by a variety of exogenous insults. Ectopic Par-4 overexpression alone is sufficient to induce apoptosis in most cancer cells, although not in normal or immortalized cells (9).

Experimental evidence indicates that Par-4 plays an important role in tumor cell survival and may be considered a candidate for tumor cell selective therapy (10). This selective ability of Par-4 to directly cause apoptosis in most cancer cells is associated with its phosphorylation state and nuclear translocation (9,11). Par-4 is phosphorylated and activated by protein kinase A (PKA), which has elevated activity in cancer cells relative to normal cells (11). Consistent with its tumor suppressor function, Par-4 is downregulated in different types of cancers, such as neuroblastoma (12), endometrial cancer (13), renal cell carcinoma (14), breast cancer (15). Par-4 knockout mice develop spontaneous tumors in various tissues (16).

Par-4 acts in both intrinsic and extrinsic apoptotic pathways. The mechanisms of Par-4 apoptosis induction involve activation of the Fas-FADD-caspase-8 apoptotic pathway, including the translocation of Fas/FasL to the plasma membrane and inhibition of the NF-κB pro-survival pathway (10,17). Studies indicate that Par-4 also functions as a transcriptional co-repressor that interacts with WT1, leading to the inhibition of BCL-2 transcriptional regulation (18). Recently, it was observed that Par-4 protein is also spontaneously secreted by normal and cancer cells to the extracellular compartment (cell culture-conditioned medium or circulating in serum) and is able to induce cancer cell-specific apoptosis by interacting with the cell-surface receptor GRP78 and activating the FADD/caspase-8/caspase-3 pathway (19). Moreover, transgenic mice that express systemic Par-4 protein are resistant to the growth of non-autochthonous tumors and intravenous injection of recombinant Par-4 inhibits metastasis (20). In cholangiocarcinoma cells, cell proliferation is associated with reduced Par-4 expression as cells selectively silenced for Par-4 demonstrated a significant increase in cell proliferation and reduced apoptosis (21). Recently, we have demonstrated that the mitogenic factors E2 and insulin-like growth factor-1 (IGF-1) lead to inhibition of Par-4 expression, suggesting that this downregulation contributes to breast cancer cell survival (22). To date, there is no report in the literature on the role of Par-4 in modulating breast cancer cell proliferation and apoptosis. The present study aimed to evaluate the effects of increased or decreased Par-4 levels on the proliferation and apoptosis of breast cancer cells. Our results demonstrate for the first time that increased Par-4 expression decreases cell proliferation rates in MCF-7 cells compared with control cells and that specific Par-4 silencing by RNAi leads to increased MCF-7 cell proliferation. Our findings also suggest that Par-4 expression increases the sensitivity of MCF-7 cells to docetaxel.

## Materials and methods

### Cell culture

Human MCF-7 breast cancer cells were purchased from the American Type Culture Collection (ATCC) and maintained in RPMI-1640 medium (Sigma Chemical Co., St. Louis, MO, USA) supplemented with 100 U/ml penicillin, 100 *μ*g/ml streptomycin, 0.25 *μ*g/ml fungizon and 10% fetal bovine serum (FBS) (Invitrogen Life Technologies, Carlsbad, CA, USA) in a humidified atmosphere of 5% CO_2_ at 37°C. For assays involving drug treatment, cells were grown in medium supplemented with 5% FBS until they reached 60–70% confluence and the medium was replaced with RPMI-1640 medium without phenol red and supplemented with 5% FBS containing 5 or 100 nM docetaxel (Taxotere, Sigma Chemical Co.) and cells were cultured for an additional 24 h.

### Plasmid transfection and clone selection

MCF-7 cells were stably transfected with plasmid pcCMV6-XL6-Par-4 expressing a full length Par-4 cDNA, or empty vector (pcCMV6-XL6-Neo) acquired from OriGene (Rockville, MD, USA). Transfection assays were performed in 6-well plates using TurboFectin 8.0 transfection reagent according to a standard protocol (OriGene). Twenty-four hours after transfection, MCF-7 cells were selected with geneticin 3000 *μ*g/ml (Gibco, Gaithersburg, MD, USA). The selected clones were maintained in 800 *μ*g/ml geneticin and screened for Par-4 mRNA expression by real-time PCR, or for Par-4 protein by western blotting.

### RNA interference to achieve PAR-4 knockdown

The duplex RNA oligonucleotides for Par-4 and silencer-negative control (scramble) were purchased from IDT^®^ (TriFECTa™ kit, IA, USA; siSeq1, HSC.RNAI.N002583.12.1; siSeq2, HSC.RNAI. N002583.12.2; and siSeq3, HSC.RNAI.N002583.12.3). In brief, 1×10^5^ cells were seeded and cultured in 6-well plates until they reached 30–50% confluence and transiently transfected with the specific Par-4 siRNA or scrambled oligonucleotides using TurboFectin 8.0 according to the manufacturer’s instructions (OriGene). Cells were harvested 24–120 h after transfection and screened for Par-4 mRNA by real-time PCR, or for Par-4 protein content by western blotting. Twenty-four hours after transfection, cells were harvested and plated in 96-well plates for cell proliferation assays using MTT, or seeded in 6-well plates for flow cytometric apoptosis assays.

### Quantitative RT-PCR

For quantitative RT-PCR analysis, cells were harvested and total RNA was extracted using the acid guanidinium thiocyanate-phenol-chloroform method. cDNA synthesis was performed using the High Capacity cDNA Archive kit (Applied Biosystems, Warrington, UK). Real-time PCR (qPCR) was performed using the Power SYBR Green kit (Applied Biosystems, Foster City, CA, USA) following the manufacturer’s recommendations. qPCR reactions were processed in the GeneAmp 7500 Sequence Detection System (Applied Biosystems) under the following conditions: 50°C for 2 min and 95°C for 10 min, followed by 40 cycles of 95°C for 15 sec and 55°C for 1 min. The PCR primers used were: for *PAR-4*, forward 5′-CCAGAGAAGGGCAAGAGCTCGG-3′; reverse 5′-ATTGCATCTTCTCGTTTCCGC-3′; *GADPH*, forward 5′-CCTCCAAAATCAAGTGGGGCG-3′; reverse 5′-GGGGCAGAGATGATGACCCTT-3′; BCL-2, forward 5′-CGACTTCGCCGAGATGTCCAG-3′; reverse 5′-CCGTCC CTGAAGAGCTCCTCC-3′; *BAX*, forward 5′-GGCCGG GTTGTCGCCCTTTTC-3′; reverse, 5′-GTCCAGCCCATG ATGGTTCTG-3′; *BID*, forward 5′-AACTCCTGTGACCAC AACATG-3′; reverse 5′-CAGGAAAGCATCTGGTAA GAA-3′; *BECLIN* forward, 5′-ACTTTCCAGAGCTACAAC ATG-3′; reverse 5′-GTCCATGGGGTTAAGAATCAA-3′. Relative expression was calculated by 2^−ΔΔCT^ (CT = fluorescence threshold value; ΔCT = CT of the target gene − CT of the reference gene (GADPH); ΔΔCT = ΔCT of the target sample − ΔCT of the reference sample). The average value of the control cells served as the reference sample. The results were expressed as n-fold differences in mRNA expression relative to expression of GAPDH and the reference sample.

### Western blot analysis

Cells were harvested and total cell lysates were prepared in lysis buffer (50 mM Na pyrophosphate, 50 mM NaF, 5 mM NaCl, 5 mM PMSF, 100 mM Na_3_VO_4_), followed by centrifugation at 13,000 rpm for 15 min at 4°C. Thirty micrograms of protein lysate were separated on a 10% SDS-PAGE gel and blotted onto nitrocellulose membranes (Pierce Biotechnology, Rockford, IL, USA). Blots were incubated with anti-Par-4 monoclonal antibody (Abcam, Cambridge, MA, USA), anti-p-ERK, anti-ERK (Santa Cruz Biotechnology, Santa Cruz, CA, USA) and anti-β-actin mouse monoclonal antibody (Millipore, Temecula, CA, USA) for 2 h at room temperature. Membranes were washed in TBS-T (25 mM Tris, 125 mM NaCl and 0.1% Tween-20) and incubated with appropriate peroxidase-conjugated secondary IgG antibody for 2 h at room temperature. Incubations were performed in 5% skim milk diluted in TBS-T. Specific proteins were detected using an enhanced chemiluminescence system (ECL^™^ Western Blotting Detection Reagents, GE Healthcare, Buckinghamshire, UK) and exposed to Hyperfilm ECL film (GE Healthcare).

### Cell proliferation assay

Cell proliferation and viability were measured using a 3-(4,5-dimethylthiazol-2yl)-2,5-diphenyltetrazolium bromide (MTT, Molecular Probes, Invitrogen) assay following the manufacturer’s instructions. Cells were seeded in 96-well plates (1×10^4^ cells/well) and maintained in RPMI-1640 medium without phenol red supplemented with 5 or 0.5% FBS. Cell growth was assessed at 0, 48, 72, 96 and 144 h. At the end of incubation, the absorbance was measured at 570 nm using the Biotrak II Plate reader (Amersham Biosciences, Cambridge, UK).

### Imunocytochemistry

MCF-7 cells were cultured in 8-well chamber slides in RPMI-1640 medium without phenol red and supplemented with 5% FBS or 5% dextran-coated charcoal-treated FBS (stripped serum, ST) for 48 h before treatment. For immunocytochemistry, cells were fixed using 4% paraformaldehyde and permeabilized with 0.5% Triton X-100. Next, cells were incubated with primary mouse monoclonal anti-Par-4 antibody 1:50 (Santa Cruz, Biotechnology; catalog sc-1666) and rabbit polyclonal anti-tubulin antibody 1:300 (OriGene), followed by the appropriate secondary antibodies conjugated with Alexa Fluor 546 and Alexa Fluor 488 1:300 (Invitrogen, OR, USA). Nuclei were counterstained with Hoechst 33342 1:3000 (Invitrogen). Cells were visualized with Carl Zeiss LSM 510 Meta (Oberkochen, Germany) laser scanning confocal microscopy. All immunofluorescence images were recorded at magnification ×20 and ×63.

### Cell cycle analysis

Floating and adherent cells were collected, washed once with PBS, fixed with 70% ice-cold ethanol and stored at −20°C until analysis by flow cytometry. The fixed cells were washed twice with PBS, resuspended in PBS containing 200 *μ*g/ml RNase A, 0.1% Triton X-100 and 20 *μ*g/ ml propidium iodide (PI) and immediately analyzed by fluorescence-activated cell sorting using the Becton-Dickinson FACSort flow cytometer (BD Biosciences, NJ, USA). The cell cycle analysis program CellQuest (BD Biosciences) was used to determine the percentage of cells at different stages of the cycle (G0-G1, S-G2/M) and the percentage of cell death using sub-G1 cells.

### Cell death analysis: Annexin V and acridine orange staining

Apoptosis was evaluated in MCF-7-pcPar-4 and MCF-7-pcNeo cells before and after treatment with 5 or 100 nM docetaxel for 24 h using the Annexin V-FITC Apoptosis Detection Kit I (BD Pharmigen, CA, USA), following the manufacturer’s instructions. Cytometric analyses were performed using the Attune^®^ Acoustic Focusing Cytometer (Life Technologies, Foster City, CA, USA). Apoptosis was also analyzed using a double-fluorescence staining technique with Hoechst 33342 and acridine orange (AO). Briefly, transfected MCF-7 cells treated with docetaxel (5 or 100 nM) for 24 h were co-stained with AO (8.5 *μ*g/ml) and with nuclear dye Hoechst 33342 (5 *μ*g/ml) for 10 min in the dark and examined under magnification ×20 and ×63 using a fluorescence microscope. Viewed by fluorescence microscopy, viable cells appear to have an intact and bright green nucleus, while apoptotic cells exhibit a bright green nucleus in which chromatin condensation is observed as dense green areas. Apoptotic cells were calculated as the ratio of apoptotic cells (with characteristic apoptotic morphology) to total cells. At least 10 fields were counted for each treatment, using the ImageJ Launcher software (Image Processing and Analysis in Java, National Institute of Health, USA).

### Statistical analysis

Statistical analyses were performed by Student’s and Mann-Whitney tests using Statistical Package for the Social Sciences 20.0 (SPSS Inc., Chicago, IL, USA). P-values were considered statistically significant at P<0.05. All data are presented as the mean ± SD of three or more replicate experiments.

## Results

### Par-4 expression, cell proliferation rates and ERK phosphorylation

**Figure 1.**
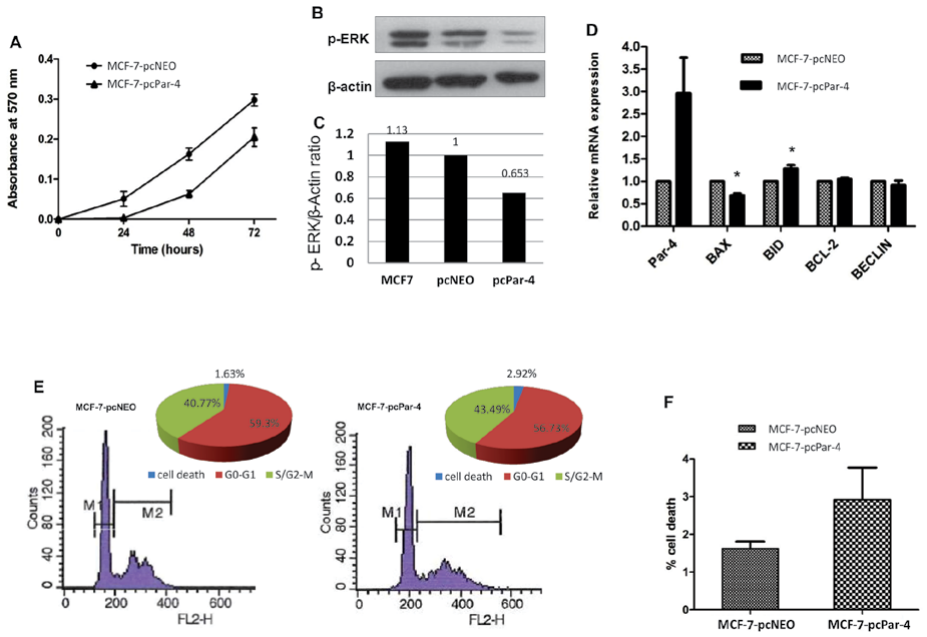
Increased Par-4 protein expression inhibited cell proliferation and ERK phosphorylation in MCF-7 breast cancer cells. (A) Cell proliferation and viability were measured by MTT assay in MCF-7-pcPar-4 and MCF-7-pcNeo cells (1×10^4^ cells) seeded in 96-well plates and maintained in normal serum (NS) for 24, 48 and 72 h. Experiments were performed in triplicate and the values are given as mean absorbance (OD 570 nm) with SD. (B and C) Cellular pERK protein levels were determined by western blot and densitometric analysis, respectively. (D) Relative PAR-4, BAX, BID, BCL-2 and BECLIN mRNA expression were determined by qRT-PCR, normalized to GAPDH. Bar height represents gene expression in cells maintained in NS relative expression in control cells (MCF-7-pcNeo). All data are expressed as mean ± SD of three experiments. ^*^Statistically significant differences, P≤0.05. (E) Flow cytometric analysis of MCF-7-pcPar-4 or control MCF-7-pcNeo using propidium iodide (PI) staining. (F) Proportion of cell death, evaluated by the cell population in sub-G1.

To evaluate the possible effects of Par-4 expression on cell proliferation, MCF-7 cells were transfected with expression vector pCMV6-XL6-Par-4 (MCF-7-pcPar-4) or with the empty vector pCMV6-XL6-Neo (MCF-7-pcNeo). Clones were selected with geneticin and were characterized by qPCR and western blotting. MCF-7-pcPar-4 tranfectants exhibiting increased Par-4 protein expression compared with MCF-7-pcNeo transfectant cells showed no significant morphological differences. Cell proliferation was significantly inhibited in MCF-7 cells expressing increased Par-4 compared with control MCF-7-pcNeo transfectants (Fig. 1A). Because the Ras/Raf/MEK/ERK pathway is a central signaling cascade involved in cell proliferation (23), we evaluated whether Par-4 might affects the status of ERK phosphorylation. Compared with control cells (MCF-7 or MCF-7-pcNeo), MCF-7-pcPar-4 cells with increased Par-4 expression exhibited significantly reduced ERK phosphorylation (Fig. 1B and C), suggesting that the proliferation restraint promoted by Par-4 involves inhibition of the Ras/Raf/MEK/ERK pathway.

We also examined whether increased Par-4 expression affected the expression profile of genes involved in apoptosis (BAX, BID, BCL-2) and autophagy (BECLIN). MCF-7-pcPar-4 cells exhibited slightly reduced BAX expression and marginally increased BID expression relative to the control cells (Fig. 1D). No detectable alterations were observed for BCL-2 and BECLIN transcripts expression (Fig. 1D).

Par-4 overexpression did not affect the cell cycle profile (Fig. 1E). However, MCF-7 cells overexpressing Par-4 display a 1.8-fold greater proportion of cells in the sub-G1 population (2.92%) compared with control cells (1.63%) (Fig. 1F).

### Selective silencing of Par-4 expression, cell proliferation and ERK phosphorylation

**Figure 2.**
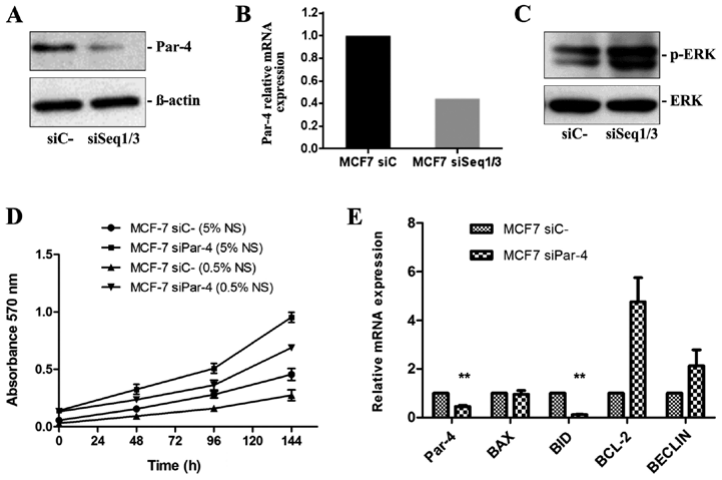
Effects of PAR-4 knockdown on cell proliferation and ERK phosphorylation in MCF-7 breast cancer cells. (A) MCF-7 cells were transiently transfected with 5 nM PAR-4-specific siRNA (siSeq1 and siSeq3), or with negative control siRNA (siC). Par-4 protein was evaluated by western blot analysis. (B) mRNA expression was determined by qRT-PCR analysis. (C) Cellular phosphorylated ERK (p-ERK) levels were determined by western blot analysis. ERK was detected as the loading control. (D) Cell proliferation and viability were measured by MTT assay in MCF-7 cells transfected with PAR-4-specific siRNA or scrambled siRNA seeded in 96-well plates (1×10^4^ cells/well) and maintained in normal serum (5% NS) or in deprived serum (0.5% NS) for 48, 96 and 144 h. The experiments were performed in triplicate and the values are given as mean absorbance (OD 570 nm) with SD. (E) Relative PAR-4, BAX, BID, BCL-2 and BECLIN mRNA expression were determined by qRT-PCR, normalized to GAPDH. All data are expressed as mean ± SD of three experiments. ^*^Statistically significant differences, P≤0.05.

To better characterize the effects of Par-4 on breast cancer cell proliferation, MCF-7 cells were transiently transfected with specific siRNA duplexes. Three different siRNA duplexes synthesized by TriFECTA were tested for Par-4 knockdown efficiency. Two Par-4 siRNAs (named siSeq1 and siSeq3) efficiently reduced Par-4 protein and mRNA expression in MCF-7 breast cancer cells compared with the control scramble siRNAs (Fig. 2A and B).

The following experiments were performed with MCF-7 cells transfected with a mixture of siSeq1 and siSeq3 siRNAs and control cells transfected with control siRNA. Western blot analysis revealed that reduced Par-4 expression leads to increased ERK phosphorylation (Fig. 2C), which is in agreement with the increased proliferation rate observed in MTT assays. MCF-7 cells displaying reduced Par-4 expression after siRNA knockdown exhibited higher proliferation rates, both in normal serum (5% SN) and when serum-deprived (0.5% NS), compared with MCF-7 cells transfected with control siRNA (Fig. 2D).

We also evaluated the effect of Par-4 knockdown on expression of the pro-apoptotic genes BAX and BID, the anti-apoptotic gene BCL-2 and the pro-autophagic gene BECLIN. Compared with MCF-7 cells transfected with scrambled siRNA, we observed significantly decreased BID mRNA expression (8.33-fold, P≤0.003) and significantly increased BCL-2 protein expression (4.76-fold) in MCF-7 cells transfected with Par-4-specific siRNAs (Fig. 2E). MCF-7 cells with reduced Par-4 expression exhibited increased BECLIN mRNA expression (2.1-fold) relative to control cells (Fig. 2E).

### Par-4 expression, apoptosis and sensitivity to docetaxel

**Figure 3.**
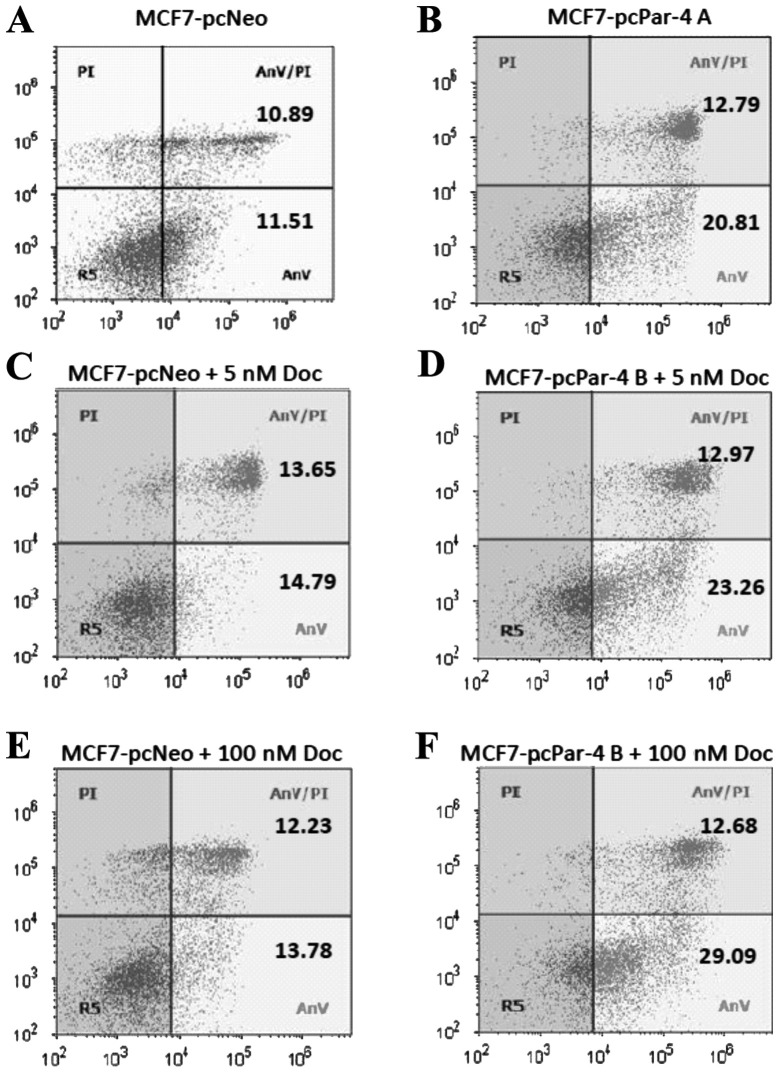
Increased Par-4 expression results in induction of early apoptosis and increased sensitivity to docetaxel. MCF-7-pcPar4 and MCF-7-pcNeo cells were treated with docetaxel for 24 h, harvested, stained with Annexin V-FITC (AnV) and/or propidium iodide (PI) and analyzed by flow cytometry. The proportion of cells positive for AnV and negative for PI (early apoptosis) and positive for both are shown in each panel.

Par-4 overexpression has been associated with increased sensitivity to apoptotic stimuli in different cell types (7). To evaluate the effect of Par-4 expression on apoptosis induction and drug sensitivity, MCF-7-pcPar-4 and MCF-7-pcNeo cells were evaluated by flow cytometry using PI/Annexin V-FITC double staining before and after docetaxel treatment (5 or 100 nM for 24 h). MCF-7-pcPar-4 cells with increased Par-4 expression exhibited a greater proportion of early apoptotic cells (stained with Annexin V-FITC) compared with control cells, in medium containing vehicle (20.81 versus 11.51%) (Fig. 3A and B), in cells treated with 5 nM docetaxel (23.26 versus 14.79%) (Fig. 3C and D) and in cells treated with 100 nM docetaxel (29.09 versus 13.78%) (Fig. 3E and F). After treatment with 100 nM docetaxel, 41.77% of cells with increased Par-4 underwent apoptosis compared with 26.01% of MCF-7 pcNeo cells.

**Figure 4.**
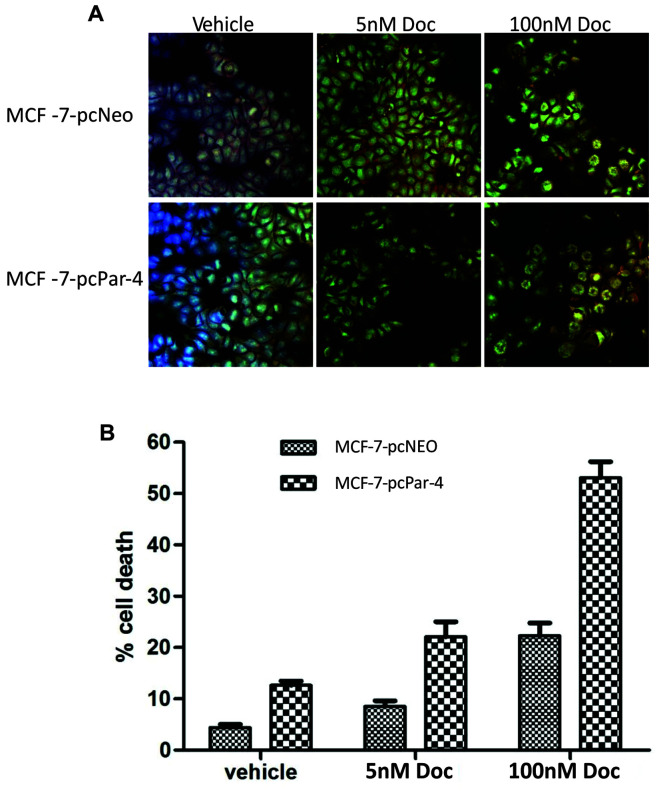
Increased Par-4 expression enhances the sensitivity of MCF-7 breast cancer cells to docetaxel. MCF-7-pcPar-4 and MCF-7-pcNeo cells were cultured in 8-well chamber slides, treated with low (5 nM) and high (100 nM) doses of docetaxel for 24 h, fixed in 4% paraformaldehyde and co-stained with acridine orange (AO) and Hoechst 33342. (A) Nuclear morphology was examined by fluorescence microscopy. (B) For quantification, attached cells stained with AO displaying abnormal nuclear morphology from 10 different areas of the well were counted using ImageJ software. Data are expressed as mean ± SD of three experiments. ^*^Statistically significant differences, P≤0.05.

A semi-quantitative analysis of the number of apoptotic cells was also performed by AO/Hoechst double labeling. MCF-7-pcPar-4 cells displayed a higher proportion of AO/ Hoechst double-stained cells exhibiting condensed chromatin, nuclear fragmentation, nucleolar disappearance, increased nuclear fluorescence and the appearance of granular apoptotic bodies compared with control cells (MCF-7-pcNeo) with vehicle (12.61 versus 4.35%, P<0.00001) and in low and high concentrations of docetaxel (5 nM: 22.09 versus 8.54%, P=0.0004; 100 nM: 53.06 versus 22.28%, P<0.0001) (Fig. 4).

## Discussion

The present study provides evidence that Par-4 plays an important role in cell proliferation and survival in breast cancer cells. Our findings indicate that elevated levels of intracellular Par-4 can sensitize MCF-7 cells to apoptosis and growth inhibition by docetaxel, which is a fundamental and effective chemotherapeutic agent against primary and advanced breast cancer, suggesting that intracellular Par-4 are involved in the pro-apoptotic and growth inhibitory action of docetaxel in breast cancer cells.

Par-4 acts in both intrinsic and extrinsic apoptotic pathways. PAR-4 induces apoptosis as a result of its ability to activate the Fas-FasL-FADD-caspase-8 pathway; by inhibiting the NF-κB survival pathway, which requires PKA-mediated PAR-4 phosphorylation at residue T155; and by downregulating Bcl-2 expression (11,17,24–26). Chan *et al* (27) reported that Par-4 mRNA and protein levels rapidly and progressively increase after trophic factor withdrawal in cultured rat hippocampal neurons and that Par-4 acts early in apoptosis (i.e., before mitochondrial dysfunction, caspase activation and nuclear changes). Previous results from our group also showed that withdrawal of estrogens and growth factors from the serum led to significantly increased Par-4 expression compared with the expression observed in MCF-7 cells maintained in media supplemented with 5% FBS (22). Furthermore, our group has demonstrated that Par-4 expression is negatively regulated by IGF-1 and the hormone 17β-estradiol (22). These findings are in agreement with results from another study, in which cholangiocarcinoma cells cultured in the absence of serum exhibited Par-4 protein increased expression associated with a significant increase in BAX protein (21).

In the present study, we used RNAi to reduce Par-4 expression and the expression vector pCMV6-XL6-PAR4 to increase Par-4 expression in MCF-7 cells. MCF-7 cells with reduced Par-4 expression exhibited faster proliferation rates relative to control cells, as well as an increase in phosphorylated extracellular signal-regulated kinases-1,2 (p-ERK) protein expression. Conversely, MCF-7 cells with increased Par-4 expression exhibited slower proliferation and decreased p-ERK protein expression compared with control cells. ERK kinase participates in the Ras/Raf/MEK/ERK signaling pathway, which is activated by mitogens and growth factors (28). ERK can phosphorylate, directly or indirectly, many transcription factors involved in cell cycle control, especially in the activation of proliferation and cell survival (including Ets-1, c-jun, c-myc and NF-κB) (23,29). In addition, components of the ERK-mediated pathway are known to be mutated or aberrantly expressed in human tumors, including breast cancer (23). These results indicate, for the first time, a possible role for Par-4 in the proliferation of breast cancer cells through the ERK1/2 pathway. The involvement of Par-4 in proliferation has also been demonstrated in cholangiocarcinoma cells (21). Specific Par-4 silencing by siRNA-activated cholangiocarcinoma cell line proliferation demonstrated a significant increase in proliferating cellular nuclear antigen protein expression, indicative of induced cell proliferation.

The primary function of Par-4 is related to its ability to increase cell sensitivity to apoptotic stimuli or direct induction of apoptosis (30,31). When we analyzed the expression pattern of apoptosis-related genes, we found that Par-4 downregulation led to increased BCL-2 and a significant reduction in BID transcripts. A member of the sub-class BH3-only, thus possessing only a BCL2 homology-3 (BH3) domain of the four BCL2 family domains (BH1, BH2, BH3 and BH4), Bid protein is a key protein in the apoptotic process, because it connects the extrinsic pathway activated by pro-apoptotic and pro-inflammatory cytokines to the intrinsic mitochondrial pathway (32,33). When cleaved and activated by caspase-8, the Bid protein can in turn cleave downstream caspases, such as caspase-3 and -7, to execute cell death; alternatively, activated Bid protein interacts with Bax and Bak, inducing permeabilization of the mitochondrial membrane to release cytochrome *c*. Thus, Bid acts by amplifying the apoptotic signal by also activating the mitochondrial pathway (34,35). Some reports in the literature have shown a relationship between Par-4 and Bid protein. A study with human prostate adenocarcinoma cells (DU145) demonstrated that cranberry extract increases Par-4 expression, which in turn activates caspase-8, leading to increased cleavage of Bid protein with consequent induction of apoptosis (36). Par-4 expression increases cleavage of caspase-8 and Bid, which translocates to mitochondria and induces the release of cytochrome *c* (37).

Interestingly, we observed a significant increase in the antiapoptotic transcript BCL-2 in MCF-7 breast cancer cells with reduced Par-4 expression, corroborating the literature regarding the inverse correlation between Par-4 and BCL-2 expression. This result suggests that silencing Par-4 protects cells from apoptosis. Qiu *et al* (25) showed that Par-4 expression leads to decreased Bcl-2 expression in NIH 3T3 fibroblasts and in PC-3 prostate cancer cells. Qiu *et al* also demonstrated that transient Bcl-2 expression prevents Par-4-dependent apoptosis in fibroblasts cultured in the absence of growth factors, indicating that the decrease in Bcl-2 is required for Par-4-induced apoptosis.

Overexpression of Par-4 in neoplastic lymphocytes decreases Bcl-2 expression, while Bax levels are not modified even with the addition of chemotherapeutic agents (26). Increased Par-4 expression sensitizes PC-3 prostate cancer cells to radiation-induced apoptosis due to inhibition of NF-κB activity, with a consequent decrease in Bcl-2 expression (38). However, it was observed that increasing Par-4 did not alter the expression of pro-apoptotic protein BAX. In this study, we also observed no significant modulation of BAX by Par-4 expression in MCF-7 cells.

Several reports have provided evidence that Par-4 acts to increase sensitivity to different chemotherapeutic drugs or chemopreventive agents. In this study, we also sought to investigate whether Par-4 might be a modulator of antitumor therapy. We observed for the first time that Par-4 sensitized MCF-7 cells to docetaxel treatment.

Docetaxel (Taxotere) and other taxanes, such as paclitaxel, are anti-microtubule drugs used as a therapeutic regimen for patients with breast, lung and ovarian cancer (39). Treatment with docetaxel inhibits key cellular events (e.g., mitotic division, secretion and transport) and triggers apoptosis and other forms of cell death (e.g., mitotic catastrophe, senescence and lytic necrosis) (40). Docetaxel may act by two mechanisms depending on its concentration in MCF-7 cells: a low drug concentration (2–4 nM) induces aberrant mitosis followed by delayed necrosis, while a high concentration (100 nM) induces prolonged cell cycle arrest (mitotic arrest) followed by apoptosis (41). Using AO staining, we observed a greater percentage of cells with morphological characteristics of apoptosis (condensed chromatin, nuclear fragmentation, nucleolar disappearance and the appearance of granular apoptotic bodies) in both low and high concentrations of docetaxel in MCF-7 cells with increased Par-4 expression.

In colon cancer cells, Par-4 overexpression led to increased apoptosis in the presence of 5-fluorouracil chemotherapy by inhibiting NF-κB activity (42). The increase in Par-4 also increased the sensitivity of Bcr-Abl-positive myeloid cells to the chemotherapeutic imatinib (tyrosine kinase inhibitor STI571) and the histone deacetylase inhibitor LAQ824 (43). Human renal cancer cells (Caki cells) overexpressing Par-4 were sensitized to apoptosis by inductor TRAIL and drugs that induce endoplasmic reticulum stress (i.e., thapsigargin, tunicamycin and etoposide) associated with decreased levels of XIAP protein and caspase activation (44). Par-4 has been shown to be secreted by both normal and cancer cells, however, it induces apoptosis only in cancer cell. Extracellular Par-4 binds to cell surface receptor GRP78 to induce apoptosis by triggering endoplasmic reticulum (ER)-stress and the caspase-8/-3 pathway (19). This apoptotic selectivity of Par-4 is due to low levels of GRP78 and lack of a robust ER-stress response in normal or immortalized cells. Our findings indicate, for the first time, that apoptosis induction by docetaxel in breast cancer cells is modulated by Par-4 expression. However, additional studies are needed to better clarify the mechanism of Par-4 and ER-stress pathway activation after treatment with docetaxel.

Taken together, these findings suggest that Par-4 expression increases the sensitivity of breast cancer cells to chemotherapeutic agents and co-parallel elevation of Par-4 may be an effective strategy to increase the efficacy of docetaxel treatment in breast cancer.

Our findings demonstrate for the first time that Par-4 modulates cell proliferation and survival in MCF-7 breast cancer cells. We show that PAR-4 inhibits MCF-7 cell growth, possibly through the inhibition of ERK-driven proliferation. Our results also show that Par-4 expression increases the rate of early apoptosis and increases the sensitivity of MCF-7 cells to docetaxel treatment. Our findings have translational relevance as they suggest that co-parallel elevation of Par-4 may increase the efficacy of breast cancer therapy.
